# Remarks upon Medicinal Inhalation, and Its Applicability as a Remedial Agent, for the Prevention and Cure of Consumption, Bronchitis, and Other Diseases of the Respiratory Organs

**Published:** 1851-11

**Authors:** Edward Jenner Coxe

**Affiliations:** New Orleans


### *Remarks upon Medicinal Inhalation, and its applicability as a remedial agent, for the prevention and cure of Consumption, Bronchitis, and other diseases of the Respiratory Organs.* By EDWARD JENNER COXE, M. D., of New Orleans.

By Medicinal Inhalation, is to be understood the inspiration of air, impregnated with the active principle of medicines, calculated to produce a local remedial effect upon that part of the mucous membrane of the respiratory organs which may be in a morbid condition, and, indirectly through it, upon those diseases implicating other portions of the lungs.

To accomplish this object in a perfect manner, two modes may be employed.

One, by causing the patient to breathe in a close room, the vapors or fumes of such medicines as may be considered appropriate to the existing disease. This is readily accomplished by placing the proper solid or fluid medicine in a vessel over a few live coals, or a lamp; an ordinary nursery lamp will admirably answer the purpose.

The other, and most efficient mode, is by means of a properly constructed inhaler, consisting of a vessel of glass, or other material, with a tightly fitting top, and two tubes, one, extending internally from the upper surface of the cover to within half an inch of the bottom, the other extending from the under surface of the cover, six or more inches in length. Through this external tube, furnished with a mouth-piece, inspiration or inhalation is to be carried on, and expiration through the nostrils. The medicinal fluid being poured into the inhaler, inhalation is effected by drawing into the lungs, through the external tube, the air contained in the inhaler over the surface of the fluid. The air thus inhaled, has its place naturally supplied from the external air by the inner tube, and passing through the medicinal fluid, is impregnated with the active principle of the medicines employed.

Such is medicinal inhalation, and as the process can be continued as long and as frequently as may be desired or ordered, it follows necessarily, that if the air does extract the medicinal properties of the articles employed, these must be applied to every portion of the mucous membrane of the air tubes, thus exerting a decided curative force.

That this effect does result, cannot admit of a doubt, and requires no reference to the numerous facts and experiments easily adduced in proof of it. The only debateable point is the selection of the appropriate remedy, or combination of remedies known, when thus applied, to exert a curative influence. By means of the inhaler thus described, the following are some of the remedies used, resulting in alleviating or curing diseases, that have proved incurable by the ordinary modes of treatment: Balsam copaiva, creosote, tar, naptha, turpentine, cyanuret of potash, iodine, iodide of potash, assafœtida, camphor, the various narcotic extracts and tinctures; tannin, galls, cayenne, in decoction and tincture, tincture of iodide of iron, the infusion or decoction of different tonics, and astringent vegetables, &c.

This manner of employing medicinal inhalation originated with Dr. Mudge, an English physician, who published a work on the subject in 1799, in which is delineated and described a correct inhaler as above.

Until the last fifteen years, the inhaler used in the United States, though called by his namc, resembled the original only in external appearance, wanting the most important part, the internal tube, to allow the passage of the external air through the medicinal fluid contained in the inhaler. This fact offers a sufficient explanation of the disappointment resulting from the employment of that misnamed inhaler, or its usual substitute, a common tea-pot. If the top of this inhaler or tea-pot be tightly fitted, it is manifestly impossible to inhale more than the limited quantity of air existing over the surface of the fluid in the inhaler; and if the top does not fit it accurately, it is equally evident that the air inspired can only be that entering from without, to supply the place of that inspired, but not passing through the medicinal fluid. In either case, it is clear, that the air not passing through the fluid, cannot contain a particle of medicinal power. Inhalation thus practised, certainly does not deserve to be noticed by those, who, in their practical works allude to medical inhalation as a means of cure or alleviation.

The reason of my personal and practical interest in this valuable remedial agent, proceeds from the fact of my having suffered for many years from a severe and constant disease of the larynx, attended by pain, hoarseness, and at times total loss of voice, and copious muco-purulent expectoration. Deriving no permanent benefit from eminent physicians of Philadelphia, or of Trousseau and others of Paris, and finding a long continued rigid system of diet, frequent leeching, cupping, bleeding, blisters, tartar emetic ointment, setons in the throat and breast, a winter's residence in the island of Madeira, and three years in Nice and other parts of the South of Europe, unable to effect a cure, I fortunately resorted to medicinal inhalation, properly so called, which succeeeded most perfectly. It may not be amiss to state, that I had previously given a most ample and long continued trial to the incorrect mode of inhalation which had been recommended, without the slightest benefit.

In consideration of these facts, would it not be strange, did I not in a decided manner assert, that medicinal inhalation does possess curative powers, and believe that the result of my experience in many cases besides my own, will not be without some weight with the profession, in reference to its intrinsic value?

A strong argument in favor of a more general and perfect trial of medicinal inhalation, is deduced from the consideration of two well established facts in reference to those prevalent and fatal diseases, consumption, bronchitis, and laryngitis, for which it is recommended as a means of prevention and cure.

The first is, that the diagnosis of the healthy or morbid condition of the respiratory organs, is at present more readily and certainly ascertained than before the discovery of the immortal Laennec.

The second, that notwithstanding this advance, the possibility of effecting a cure in confirmed cases of the above diseases, is by many considered problematical, or at least, of exceedingly rare occurrence.

The statistics of our country and Europe, prove the generally fatal termination of those diseases, regardless of the stereotyped system of treatment so pertinaciously persisted in. In the exceptional cases, where a cure does result, it may be questioned whether such is due to the medical treatment strictly speaking, with the exception of that part, having a tendency to maintain and increase the general strength, thereby enabling the restorative power of nature to heal by her own laws, those morbid conditions, depending upon many general causes, and requiring the aid of hygienic, rather than medical treatment. Numerous authorities could be cited to prove that the commencement of many diseases for which medicinal inhalation is particularly proposed, depends upon a morbid or depraved condition of the system, either hereditary, or resulting from the combined influence of poor living, impure air, and a total disregard of common sense in respect to clothing, exercise, or diet, finally localizing itself upon the important pulmonary organs, thus undermining the main springs of health, and rendering, after a time, all efforts to arrest its progress of no avail. It necessarily follows, if we are to hope for improvement in the treatment and termination of the diseases alluded to, that we must not wait until we have presented all the symptoms indicating the firm hold upon the organs, but rather watch for the forming germ, and adopt hygienic and other measures, to arrest and overcome the morbid predisposition, and force or coax nature to repair the evils resulting from a combination of causes, favorable to the rise and progress of an almost incurable disease. As one of the means, well calculated to aid in the accomplishment of so desirable an end, by giving vigor to the pulmonary organs, and others connected with them, I have no hesitation in assigning a very prominent place to medicinal inhalation.

In pursuing the consideration of this curative agent, it is necessary to enquire into its mode of action, which I regard as twofold. The one, altogether mechanical, consisting in the gradually increased expansion of the lungs, by drawing a large volume of air into them, allowing them to remain in this expanded state for as long a time as can be endured without pain or uneasiness, and finally permitting the air to be expired slowly through a valvular opening of the lips, a small reed, or quill, or one of the many breathing tubes made for that purpose, though in no respect superior to the other modes. By repeating this process more or less frequently, during the day, for fifteen or twenty minutes each time, we have an increase of size, or capacity and strength, and as an effect, a permanent enlargement and strength of the thorax, an increased action of the heart, with a more perfect distribution of the blood to all parts of the system.

The second consists in the remedial and curative power exerted by the local application of the vapor or fumes of such remedies as may be indicated by the symptoms of each case, and the stage of the disease.

By the correct adaptation of remedies to the existing disease, we can produce positive effects, similar, and often superior, to those resulting from the internal exhibition of medicines, with the advantage of not harassing the stomach and bowels.

If successful in establishing what may correctly be attributed to the local application of remedies, may it not be expected, that inhalation will be more generally brought into notice, its claims upon the profession more clearly defined, and the proper manner of using it, as well as the medicines to be employed, more specifically laid down for the guidance of those who may desire to benefit others by its employment?

In endeavoring to place medicinal inhalation upon its proper basis, let it not be supposed that impossibilities can be effected, by restoring to perfect health those already at the verge of the grave. Let it rather be regarded as a remedy to be employed at the forming stage of the disease, or, in those cases where such may be apprehended, to impart vigor to the thoracic organs, and thus arrest the progress of morbid action.

An occasional trial will not suffice to decide as to the actual value of this remedial agent; time, perseverance, and a long continued series of observations are indispensable. The end to be attained is certainly deserving of our most strenuous exertions. In the following observations and conclusions of those who have recorded their experience, it will be seen, that some allude to the mechanical action of inhalation, others to the curative effects resulting from the different medicines employed. Dr. A. T. Thompson says, "There is one description of exercise too little attended to, but which is nevertheless, of great importance in warding off pulmonary diseases, namely, the exercise of the chest. Nothing is more essential for the preservation of health, than the full expansion of the lungs, so as to maintain the free passage of the air to the minutest tubes and all the air cells, to promote the pulmonary circulation, and to favor that complete change in the blood, for which the respiratory motion is intended." Dr.

Tweedie says, "Changes of structure in the bronchial tubes, are most commonly the result of inflammation, or of some kindred modification of the nutritive process. Frequent recurrence, or long continuance of inflammation of the bronchial membranes, as in other structures, changes their condition, and the mechanical forces to which they are subjected in the junction of respiration, may variously modify this change. Inasmuch as these lesions seem to arise from continued inflammation, it becomes of the more importance to direct remedies against those forms of bronchitis, that are habitual, or frequently recurring. An imperfectly cured cough will often harass a patient for months, and even for years. In process of time, the breathing becomes permanently shortened, and an irritation is often fixed in some of the tubes, manifesting its effects on their secreting function by habitual expectoration, generally thin and mucous, sometimes muco-purulent. There is one point with regard to treatment particularly suggested by a knowledge of this change of structure—that, not only should the practitioner persevere in the use of the means which tend to eradicate the low degrees of inflammation that produce it, but he also should endeavor to countervail, by mechanical means, that mechanical limitation, which this change induces in the size of the tubes. If the patient use no exertions, and give his lungs little play, any increase in the rigidity of the tubes will more readily fix them in their present contracted state; but if he take moderate exercise, increased as habit improves his power, the lungs will be kept in that free mobile condition that is least favorable to rigidity or deposition of any kind. Probably, special efforts of inhalation would be useful with the same view, and, as this might be combined with some mildly stimulating vapor, such as that of water impregnated with tar, or camphor, it might also be serviceable in improving the secreting properties of the membrane."

Dr. Good, observes, "A moderate use of the vocal organs, as of any other, tends to strengthen them, and to enable public speakers, singers, and performers on wind instruments, to go through great exertion, without inconvenience, which would be extremely fatiguing to those who are but little practised in any of these branches; but the labor is often carried too far, and the lungs become habitually irritated, and hæmoptysis succeeds.

The organs of respiration, like those of every other kind, derive strength instead of weakness, from a temperate use of them."

Says the eminent Professor Rush, "Those persons who have been early instructed in vocal music, and who use their vocal organs moderately through life, are seldom affected by a hæmorrhage from the lungs. Lawyers, players, public criers, and city watchmen, all of whom exercise their lungs either by long or loud speaking, are less affected by this disease than persons of other occupations. The lungs, when debilitated, derive equal benefit with the limbs, or other parts of the body, from moderate exercise."

Dr. Mudge says, "The two great indications, of preventing an increased irritation by the cough, on the inflamed parts, and removing inflammation itself, by such emollient applications as could most conveniently be applied to them, are thoroughly answered by opium, and the inhaling warm steams into the lungs, and the fact is past dispute, that the conjoined powers of those agencies are a sure, and, in general, an immediate cure."

Van Swieten remarks, "It is certain that steam and vapors drawn in with the air in respiration may be of use, as they everywhere come in contact with the whole aerial cavity of the lnngs, and thus various remedies may be applied, according to the various conditions of the ulcer."

Dr. Baillie says: "In quinsy, should leeching and other treatment not materially lessen the inflammation of the tonsils, and velum palati, the progress of the inflammation should be encouraged by inhaling the vapors of boiling water and vinegar, as, by this means, the disease goes more quickly through its process, and the patient suffers less."

Dr. Pringle remarks: "I have likewise observed good effects from making the patient breathe over the steam of hot water, a practice recommended by Bœrhaave and Van Swieten, and confirmed to me by the repeated trials of Dr. Huck, who found it more beneficial when the phlegm was viscid, as well as more grateful to the patient by adding a small portion of vinegar."

Dr. Rush says in a note to the above: "Too much cannot be said in favor of this simple and powerful remedy; the editor has seen patients snatched from the jaws of death by it."

Dr. Crichton, after giving cases in which fumigations of tar had been used, remarks: "It must be evident from the preceding cases that the tar fumigations, though completely successful in some of them, did not produce the same good in all, but on the other hand, the very great relief which every patient has experienced at first from it, particularly in the diminution of cough, expectoration, and hectic fever, is a fact which ought to encourage us to multiply the trials of this remedy as far as possible."

Delpit observes: "If any species of consumption exists in which simple or compound fumigations become useful, it is certainly in that of the larynx, more accessible and susceptible to their influence."

Mascagni says: "If an efficient remedy for diseases of the lungs is ever discovered, it will be one that can be applied by inhalation."

Dr. Thomas relates a case of hooping cough of three weeks' standing, cured in ten days, by confining the patient in a room, and causing him to inhale the fumes given out by adding gradually half an ounce of powdered nitrate of potash, to half an ounce of sulphuric acid in a tea-cup.

Dr. Pearson found the vapor of ether very serviceable in cases of consumption, and that, in some cases, combined with musk, camphor, opium, assafœtida, and similar articles, it appeared to possess greater power.

Dr. Ramadge lays great stress upon the advantages resulting from the free use of inhalation through a long tube, which is altogether mechanical in its action. He says: "Inhalation performed two or three times daily, for half an hour each time, will, in the short space of a few weeks, work a wonderful change on the chest externally; the muscles concerned in respiration will be manifestly enlarged, and the bony compages of the chest visibly increased, whilst, at the same time, the natural respiratory murmur will be heard far more distinctly than ever."

Dr. Elliotson says: "I have made many phthisical patients breathe for four or five minutes, four or five times a day, through a mixture of creasote with mucilage and water, but with no further effect, in general, than occasionally an increased facility of respiration, and a diminution of the cough and expectoration. Some, it always appears to irritate, and all in whom any degree of inflammation exists. I am satisfied it is no remedy for tubercles. Where, however, only a single ulcer, or but a small number, exist in the lungs, and there is no disposition to further tubercular formation, it is very beneficial. In bronchorrhœa, or that state of the bronchial mucous membrane which consists in a profuse secretion without inflammation, I have seen its inhalation of essential service, and in one instance of this affection in which the expectoration was extremely offensive, the cure was very rapid. In asthma, also, dependent upon morbid excitability of the bronchial membrane, its inhalation is often useful."

In chronic affections of the fauces, larynx, trachea, and bronchi, I have frequently employed creosote by inhalation, generally combined with other medicines, with marked benefit, and while the testimony of Dr. E. is decidedly favorable to its use, it clearly shows the necessity of discrimination in the selection of remedies, no less than attention to the existing stage of the disease.

Dr. Mackintosh remarks: "The inhalation of hot vapors will be found very serviceable in croup, and in chronic complicated cases of bronchitis, service may be expected from the inhalation of tar vapor. In scarlet fever, when the throat is much affected, inhaling the vapor of warm water affords more ease than any argle."

Dr. Eberle says: "The inhalation of aëriform fluids may be employed to great advantage in the treatment of pulmonic affections. In this way we are enabled to make direct impressions on the respiratory organs, a circumstance which experience has shown to be of much consequence in many of the diseases to which these organs are liable. In catarrhal affections, attended with painful and difficult expectoration, much benefit may generally be obtained from the inhalation of the steam of hot water, or of vinegar and water. This acts as an emollient and soothing application to the tender and inflamed vessels of the internal surface of the bronchial tubes.

In Pneumonia, after the violence of the arterial excitement has been reduced by depletory measures, the inhalation of the steam of hot water, or decoctions of emollient herbs, will often contribute much to the support of an easy and regular expectoration. In no affection, however, are inhalations of this kind more decidedly beneficial than in the paroxysms of asthma. Inhalations of warm water and vinegar are often very serviceable in cynanche tonsillaris and trachealis. The inhalation of ethereal vapors is a remedy of very considerable value in certain affections of the respiratory organs. In dyspnœa, depending on a spasmodic condition of the pulmonary system, I have frequently derived very great benefit from the inhalation of the vapors of sulphuric ether. In two cases of hooping cough that had been mismanaged during the early period of the disease, and in which the expectoration had assumed a purulent appearance, I have derived decided benefit from tar fumigations. This remedy has also been found very useful in asthmatic affections. In acute inflammatory diseases of the lungs, however, it cannot be employed without doing mischief. The inhalation of the tar fumes appears to be particularly beneficial in chronic bronchitis, or in that form of pulmonary consumption which depends on chronic inflammation of the mucous membrane of the bronchi."

Dr. Thomas observes: "To moderate the severity of the paroxysms in asthma, we cannot employ a more powerful and efficacious means of relief than the inhalation of steam frequently from an inhalator; an infusion of chamomile flowers, with the addition of a little ether, may be used on the occasion."

Sir C. Scudamore remarks, "Much professional scepticism appears to be entertained concerning the possibility of affording any material relief in cases of consumption. This I must condemn. It is I conceive less adverse to the interest of science than of humanity, to consider any disease as absolutely incurable. Our art is doubtless bounded by certain limits, but let not these limits be still further circumscribed by our own supineness and prejudices. It has often happened, that valuable remedies have been laid aside or neglected, in consequence of some mismanagement in their use, or perhaps, from an excessive zeal in the recommendation of them by their authors, so that not being found capable of producing the promised effects, they have experienced the unmerited fate of being rejected as useless.

"Some of the medicines which I have recommended for inhalation are agents of much delicacy and power. My conviction of their most perfect safety employed in this manner, has not been shaken by a single untoward instance, but it is right to state, that their administration requires careful attention and management. The composition of an inhaling mixture, and the doses to be used, are to be adapted to the particular case, and changed according to its varying circumstances, in the same manner as we find it necessary and proper to alter and accommodate our treatment with internal medicines.

In consumption, even in desperate circumstances, I recommend the use of inhalation, as being calculated more than any other treatment to mitigate the most troublesome symptoms, and afford the patient great comfort and relief; also, I am persuaded, that such treatment affords the strongest chance of cure. Before the disease has made much destructive progress, and especially in the very early stage of phthisis, I have the highest opinion of the efficacy of the treatment. But I desire to repeat what I have before said, that internal treatment and general management should be joined with the plan of inhalations. I have occasionally, as shown in some of the cases which I have related, found the pulmonary or bronchial disease in so great a degree local, that I have chiefly or wholly trusted to the use of inhalations, and with success; but these are exceptions to the general rule. In chronic bronchitis, the benefits of inhalation are too well proved by the speedy favorable alteration produced both in the quantity and quality of the expectoration, and by the sensible relief which is experienced by the patient, that no question of the value of the remedy can be entertained. It gives relief to the asthmatic patient, proves often curable in cases of consumption, not become desperate in their nature, and is capable of much useful influence even in those extreme examples of the disease which too probably admit only of alleviation, and seem to bid defiance to the ordinary rules of practice."

Dr. Corrigan says, "Of the powerful influence which various vapors and even changes in the air itself exercise as local agents on the lungs there cannot be a doubt; every day's observation shows it; every one in his own person feels it. Even allowing most fully for the exaggerated encomiums of some of the older advocates of inhalation, enough remains in the attestations of such men as Darwin, Beddoes, Withering and their contemporaries, to forbid us to abandon this plan of treatment. I think these few observations justify me in coming to the conclusion, that remedies in the form of vapor exert a powerful influence over diseased action; and that, as it is only in this form we can ever administer remedies to act locally upon diseased tissues in the lungs, their exhibition in this form merits close attention and further perseverance."

In the Boston Medical Journal for January 1851, Dr. Cornell observes in an article on inhalations, that, following a plan of Dr. Chambers of London, he has employed the inhalation of fine powders with advantage in diseases of the air passages. The powder to be used is prepared by soaking lycopodium or club-moss in a saturated solution of nitrate of silver, then drying and reducing to a very fine powder. It is applied by placing the tubular end of a glass funnel in the mouth, and simultaneously with a deep inspiration the powder is dusted into the large end, whereby it is diffused over the back part of the throat, a portion at times entering the larynx. This must be regarded as a modification of the plan of insufflation, proposed and practised many years since by Trousseau of Paris.

After having, by his advice, tried most fairly this plan for a long time on myself, without deriving any permanent benefit, and having been three times, from its perfect application and entrance into the larynx, on the point of suffocation, I was forced to abandon its further use, satisfied that no little damage attended, provided the entrance of the powder into the larynx was desired. If this is not, I am perfectly convinced from repeated trials, that the insufflation of the powder is in no respect equal in power or general utility, to the strong solution of nitrate of silver, as recommended and applied by Dr. Green of New York.

In reference to the introduction into the larynx of a strong solution of caustic, while according to Dr. Green the entire merit of so valuable an innovation, and equally convinced in many cases of its great and peculiar value, I feel obliged to remark that, in the majority of cases of diseases of the throat, the introduction into the larynx of the sponge saturated with the caustic, will not be found of absolute necessity; cases are of frequent occurrence in which, by the application of the strong solution to the back and lower parts of the fauces, diseases of the throat have been perfectly cured, after the failure of all other modes of treatment, except that by inhalation, which had never been resorted to. By means of the strong solution of caustic, in conjunction with medicinal, inhalation, many cases of disease, incurable by other resources will be found perfectly manageable.

Dr. Cornell alludes, in terms of commendation, to another mode of using the nitrate of silver, by imbibing from the mouth of a tea-pot, the vapor arising from a solution of forty-eight grains of nitrate of silver, in eight ounces of boiling water.

To determine the possibility of producing any effect from the local application of caustic, by this, or the proper mode of inhalation, I instituted the two following experiments, using every precaution to make them correct:

After making an inhaler of glass, I poured into it two ounces of distilled water, and fifty grains of the crystallized salt, and when thoroughly dissolved, continued for ten minutes the inhalation, causing the air expired to pass into a solution of common salt. Neither on the palate nor in the solution was the slightest impression made, or effect produced.

To render the matter still more conclusive, I distilled by a glass retort, six ounces of water, and dissolving in this sixty grains of the crystals, I again distilled five ounces from this strong solution, and by testing this in various ways, it was conlusively shown that not a particle of the silver was present, and that, consequently, that mode of employing the caustic was utterly useless.

Although no additional proof was required, I swallowed three ounces of this water, without perceiving any effect as might be supposed. Firmly convinced of the efficacy of medicinal inhalation, properly performed, as a preventive and curative agent, it is to be hoped that it will be more generally resorted to, more systematically and correctly performed, and that the apathy manifested by the majority of the profession in its practical application, will yield to a determination to pursue the subject with an enthusiasm worthy of the object to be attained.

The following remarks of Sir C. Scudamore may appropriately conclude the subject: "It may be laid down as a principle, that inhalation should always be so conducted as not to produce fatigue to the patient in any way, either as regards the composition of the mixture, its strength, or the period of carrying on the process."

